# Supplementary material for: The Association between Stressful Life Events and Emotional and Behavioral Problems in Children 0–7 Years Old: The CIKEO Study
Source: Int J Environ Res Public Health. 2022 Jan 31;19(3):1650. doi: 10.3390/ijerph19031650 (PMC8835208; doi:10.3390/ijerph19031650)
Supplement: Supplementary file 1 [file ijerph-19-01650-s001.zip › ijerph-1536905-supplementary.pdf]

**Supplementary Files:** The association between stressful life events and emotional and behavioral problems in children 0-7 years old: the CIKEO study

*Corresponding author: Hein Raat, PhD, Department of Public Health, Erasmus University Medical Centre, Rotterdam, PO box 2040, 3000 CA Rotterdam, The Netherlands; Email: h.raat@erasmusmc.nl; ORCID: https://orcid.org/0000-0002-6000-7445

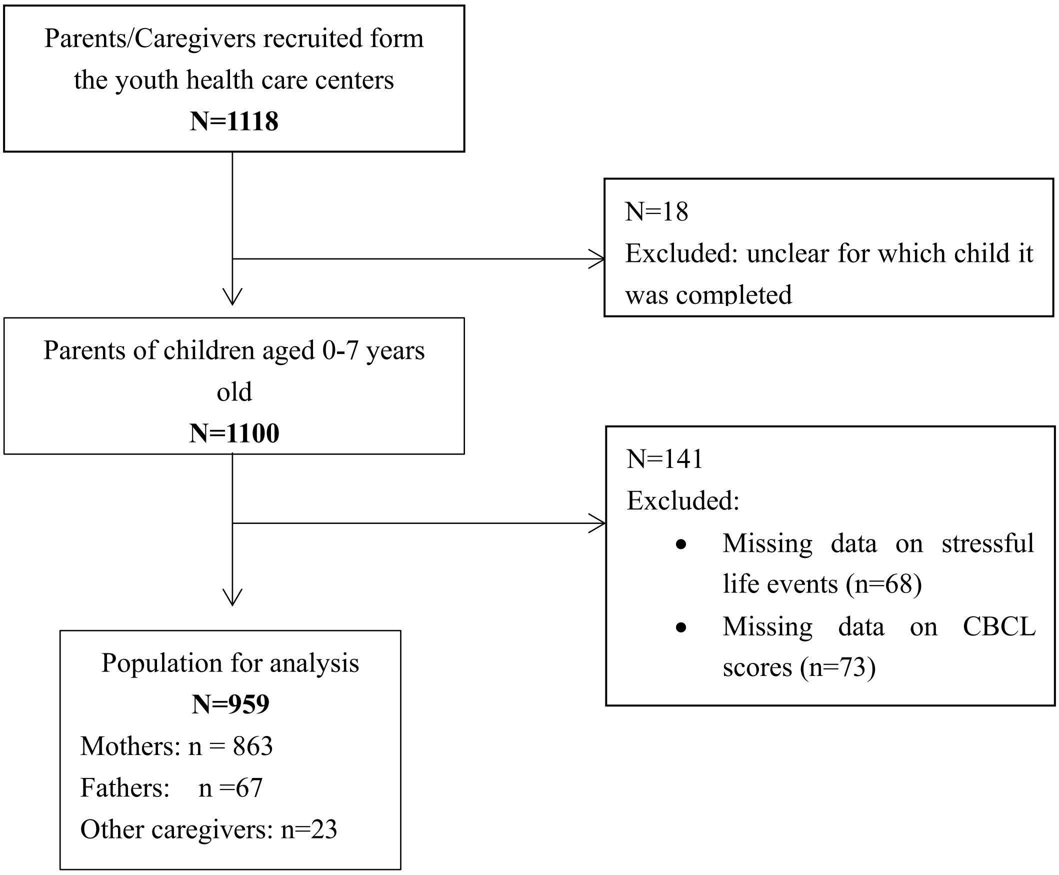


**Supplementary Figure S1.** Flowchart of participants included for analysis

**Supplementary Table S1.** Association between stressful life events (overall severity experience) and problem behaviors in children aged 0-7 years old (n=959**)**

| | CBCL-T | | CBCL-E | | CBCL-I | |
|---|---|---|---|---|---|---|
| **Overall severity experience** * | **Mean (SD)** | **β (95%CI)** # | **Mean (SD)** | **β (95%CI)** | **Mean (SD)** | **β (95%CI)** |
| No-expose (N=232,24.2%) | 16.31 (14.98) | REF. | 7.22 (6.44) | REF. | 3.79 (4.73) | REF. |
| Low (1-2, N=299, 31.2%) | 19.02 (15.49) | ***3.09 (0.56, 5.79)*** | 8.56 (7.07) | ***1.64 (0.51, 2.88)*** | 4.30 (4.67) | 0.47 (-0.27, 1.23) |
| Middle (3-5, N=246, 25.7%) | 20.59 (15.46) | ***4.13 (1.63, 7.03)*** | 8.74 (6.57) | ***1.60 (0.46, 2.83)*** | 5.11 (5.11) | ***1.26 (0.43, 2.12)*** |
| High (⩾6, N=182, 19.0%) | 27.27 (20.12) | ***10.08 (6.85, 14.08)*** | 11.52 (8.07) | ***4.20 (2.76, 5.84)*** | 6.57 (6.56) | ***2.44 (1.47, 3.66)*** |
| **P for trend** | | <0.05 | | <0.05 | | <0.05 |

Note: * Overall severity experience were calculated by summing the severity scores of all SLEs an individual experienced in the past 12 months; # derived from 2000 bootstrap samples with replacement. CBCL-T=Child Behavior Checklist Total Scale score, CBCL-E=Child Behavior Checklist Externalizing Subscale score, CBCL-I=Child Behavior Checklist Internalizing Subscale score; REF=reference; Adjusted for child age, sex, ethnic-background, respondents' age, sex, educational level, family composition, household income and recruitment methods; SLE= Stressful Life Events; bold and italic indicates p<0.05.
